# Management of indeterminate hepatic nodules and evaluation of factors predicting their malignant potential in patients with colorectal cancer

**DOI:** 10.1038/s41598-021-93339-w

**Published:** 2021-07-02

**Authors:** Mizelle D’Silva, Jai Young Cho, Ho-Seong Han, Taupyk Yerlan, Yoo-Seok Yoon, Hae Won Lee, Jun Suh Lee, Boram Lee, Moonhwan Kim

**Affiliations:** Department of Surgery, Seoul National University Bundang Hospital, Seoul National University College of Medicine, Gumi-ro 173, Bundang-gu, Seongnam-si, Gyeonggi-do 13620 Republic of Korea

**Keywords:** Surgical oncology, Colorectal cancer

## Abstract

Some liver nodules remain indeterminate despite hepatocyte-specific contrast MRI in patients with colorectal liver metastasis (CRLM). Our objective was to study the natural course and evaluate possible treatment strategies for indeterminate nodules. We retrospectively evaluated patients in whom MRI revealed ‘indeterminate’ or ‘equivocal’ nodules between January 2008 and October 2018. Patients were followed up until October 2019 or until death (median, 18 months; (1–130 months)). The incidence of patients with indeterminate nodules on MRI was 15.4% (60 of 389). The sensitivity and specificity of intraoperative ultrasound for detecting indeterminate nodules were 73.68% and 93.75%, respectively, with a positive predictive value of 96.6%. Over half of the patients followed up had benign nodules (58.8%). By comparing characteristics of patients with benign or malignant nodules in the follow up group, the ratio of positive lymph nodes to total number of lymph nodes resected (pLNR) was significantly greater in patients with malignant nodules (*P* = 0.006). Intraoperative ultrasound could be considered as an adjunct to MRI in patients with indeterminate nodules owing to its high positive predictive value. The pLNR could be used to help select which patients can undergo conservative therapy, at least in metachronous CRLM.

## Introduction

The management of colorectal carcinoma (CRC) has undergone major changes in recent years, especially in the management of metastatic CRC. The liver is the most common organ to be affected by colorectal metastasis^1^. Recently, resectability of colorectal liver metastasis (CRLM) has changed rapidly. After neoadjuvant chemotherapy combined with targeted therapy, the resectability rate has increased up to 70–90%, and concurrently 70% of unresectable patients^2,3^. The differential diagnosis of CRLM may include primary intrahepatic cholangiocarcinoma, primarily because CRC is usually an adenocarcinoma^4^.

About 15–20% of liver metastases are detected at the time of the diagnosis of the colorectal cancer and additional 35–45% of liver metastasis are newly diagnosed during the course of the colorectal cancer treatment^5^. A recent report found an association of KRAS with worse recurrence free survival (RFS) and overall survival (OS) among patients with a left-sided primary CRC^6^. In addition it was found that left-sided primary tumors were associated with improved median OS after resection of CRLM^7^.

Liver resection currently is the only potentially curative treatment for CRLM. Thus, accurate diagnosis of these lesions is of paramount importance. KRAS mutation detected in approximately 30–50% of CRC is a predictor of oncologic outcomes^8^. Analysis of the primary tumor may suggest the mutational status of CRLM^9^. The prognostic impact after hepatic resection for CRLM varies based on KRAS status and site of the primary CRC^6^.

Imaging is vital for diagnosing CRLM. Computed tomography (CT) is generally preferred for initial imaging because it is cheap, quick, and widely available. However, with the advent of tissue-specific contrast agents, magnetic resonance imaging (MRI) is increasingly being used to diagnose small lesions that are not easily characterized on CT. In addition, the CT image is may also be compromised in patients who have received chemotherapy due to sinusoidal dilatation and injury caused by chemotherapeutic agents, interfering with the attenuation of hepatic parenchyma^10^.

It has been reported that small, indeterminate liver lesions may occur in up to 16.7% of patients with CRC^11^. However, even with the use of hepatocyte-specific contrast agents, some nodules may remain indeterminate or new indeterminate nodules may be identified by MRI. Currently, there are no established clinical criteria or strategies for managing these nodules. Therefore, the objective of this study was to observe the natural course of indeterminate hepatic nodules detected on MRI and evaluate appropriate management strategies for these lesions.

## Methods

The study was approved by the institutional review board at Seoul National University Bundang Hospital, Seongnam, South Korea. All methods were performed in accordance with the relevant guidelines and regulations. Informed consent was obtained from all the participants in the study. CRC patients treated with chemotherapy primarily underwent surgery for CRC along with liver resection if synchronous metastasis were present. In case of just a primary colorectal tumor, surgery was done and liver metastasis was detected on routine follow-up. A total of 473 patients admitted to the hospital with either synchronous or metachronous CRLM were assessed for inclusion in this retrospective study. All patients underwent a routine CT scan for screening, and patients with CRLM detected by CT were further evaluated with a Gadoxetic acid MRI (Primovist®, Germany). Of the 389 patients who underwent MRI, 60 patients with indeterminate or equivocal nodules detected by gadoxetic acid-enhanced MRI between January 2008 and October 2018 were included in the present study. Patients whose MRI reports stated ‘most likely malignant’ or ‘most likely benign’ were excluded from the study. All the patients were followed up until October 2019, with a median of 18 months (range 1–130 months).

The Eastern Cooperative Oncology Group (ECOG) scale was used to evaluate performance status (PS) of patients. Abdomino- pelvic CT scans were performed as part of the routine follow-up protocol with an interval of every 3 months or less. Gadoxetic acid-enhanced liver MRI was additionally performed if there was a new hepatic lesion or substantial interval growth of the previously noted equivocal lesion to assess resectability. Treatment response was assessed in accordance with the response evaluation criteria in solid tumor (RECIST) version 1.1 (Eisenhauer et al. 2009)^2,12^.

Standard abdominal ultrasound was not performed before the surgery. For both open and laparoscopic surgery, the surgeons mobilized and evaluated the liver by inspection and/or palpation. In addition the surgeons or radiologists who had full knowledge of the preoperative imaging findings performed intraoperative liver ultrasonography (SSD-3500, Aloka, Japan; MylLab 25 Gold, Esaote Biomedica, Italy; or iU22, Philips Medical Systems, The Netherlands) to detect new lesions and further characterization of small indeterminate nodules^13^. Any metastatic nodules identified on intraoperative liver ultrasound (IOUS) were either resected or ablated. The nodules that could not be detected by IOUS were followed up, except for any that were unintentionally resected as part of a larger surgical specimen.

Recurrence was defined as radiological or pathological confirmed recurrence at the site of the previous indeterminate nodule. Sensitivity and specificity were calculated using the number of true positives, false positives, true negatives and false negatives, True positives and true negatives were taken as the number of tumors identified on IOUS which were confirmed as positive or negative on pathology. False negatives and false positives were taken as patients where the IOUS findings and pathological findings differed. Survival was calculated from the date of resection to the date of last follow-up or death.

Statistical analysis was performed using SPSS for Windows version 20 (Chicago, Illinois, USA). Categorical data were expressed as numbers and percentages. Continuous data were expressed as mean ± Standard deviation. Univariate analysis was carried out using the χ^2^ test.

## Results

### Patient characteristics

Indeterminate nodules were detected by MRI in 60/389 (15.4%) patients, which included 43 (71.7%) males (Table 1). The mean age of the patients was 61 years (range, 36–82 years). Among these 60 patients, 43 (71.7%) had solitary indeterminate nodules, 36 (60%) had synchronous lesions, and 24 (40%) had metachronous CRLM. We routinely performed PET scan for all patients with colorectal cancer. However, there was usually no uptake from PET scan in patients with small indeterminate liver nodules, especially nodules less than 5 mm of size.

**Table 1:** Patient characteristics.

Characteristics	Values n (%)	Mean + SD
Age		61.55 ± 9.95
Male	43(72)	
Laparoscopy	13(22)	
Anatomical liver resection	26(43)	
Lymphatic invasion	34(57)	
Perineural invasion	38(63)	
Venous invasion	27(45)	
Colon cancer	39(65)	
Rectal cancer	21(35)	
Synchronous lesions	36(60)	
No of nodules on MRI		3.93 ± 2.76
Neoadjuvant chemotherapy	16(27)	
Adjuvant chemotherapy	49(82)	
CEA at diagnosis of metastasis		27.68 ± 71.37
Size of indeterminate nodules		0.7 ± 0.45
Recurrence	16(27)	
T1	2 (3)	
T2	3 (5)	
T3	38 (64)	
T4	17 (28)	
N0	9 (15)	
N1	21 (35)	
N2	29 (48)	

*MRI* magnetic resonance imaging, *CEA* carcinoembryonic antigen.

The most common histologic grade of primary CRC was moderately differentiated. The primary CRC was located in the colon in 65.0% of patients and in the rectum in 35.0%. The T stage of the primary was mostly T3 or above. The number of resected nodes ranged from 3 to 117. For primary CRC, lymphatic invasion was found in 56.7%, perineural invasion in 63.3%, and venous invasion in 45.0% of patients.

### Results of gadoxetic acid-enhanced MRI

The number of nodules detected by MRI ranged from one to 12. MRI revealed at least five nodules in 12 (20.0%) patients. Neoadjuvant chemotherapy was administered to 16 (26.7%) patients before liver resection, while 49 (81.7%) received adjuvant chemotherapy after liver resection. Malignancy was detected in 9 (56.3%) of patients who received neoadjuvant chemotherapy. The mean carcinoembryonic antigen level at diagnosis was 27.7 ng/mL (range 1–520 ng/mL). The mean size of the indeterminate nodules was 0.7 cm (range 0.2–1.5 cm).

### Diagnostic accuracy of IOUS

The sensitivity and specificity of IOUS for detecting malignant indeterminate nodules were 73.68% and 93.75%, respectively. The positive predictive value was 96.6%. Patients whose nodules were ablated (10%) were excluded from this analysis of diagnostic accuracy.

### Natural history of small equivocal lesions detected by MRI (Fig. 1)

**Figure 1:**
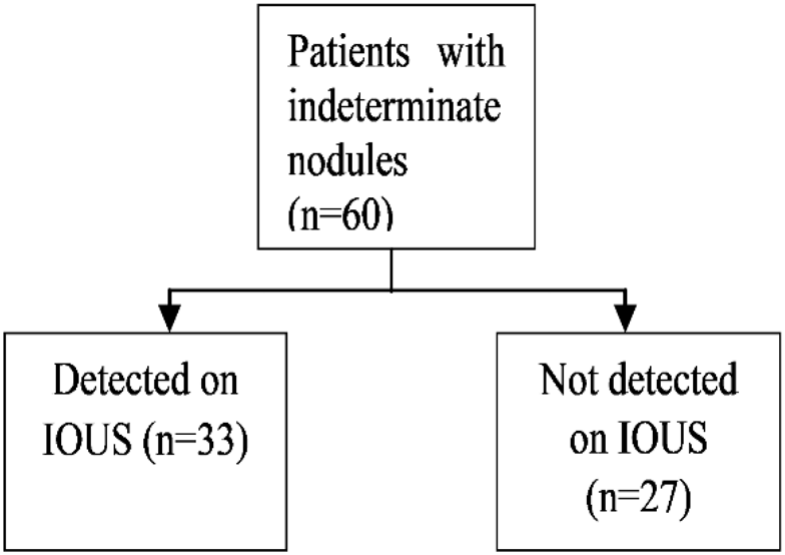
IOUS for indeterminate nodules.

**Figure 2:**
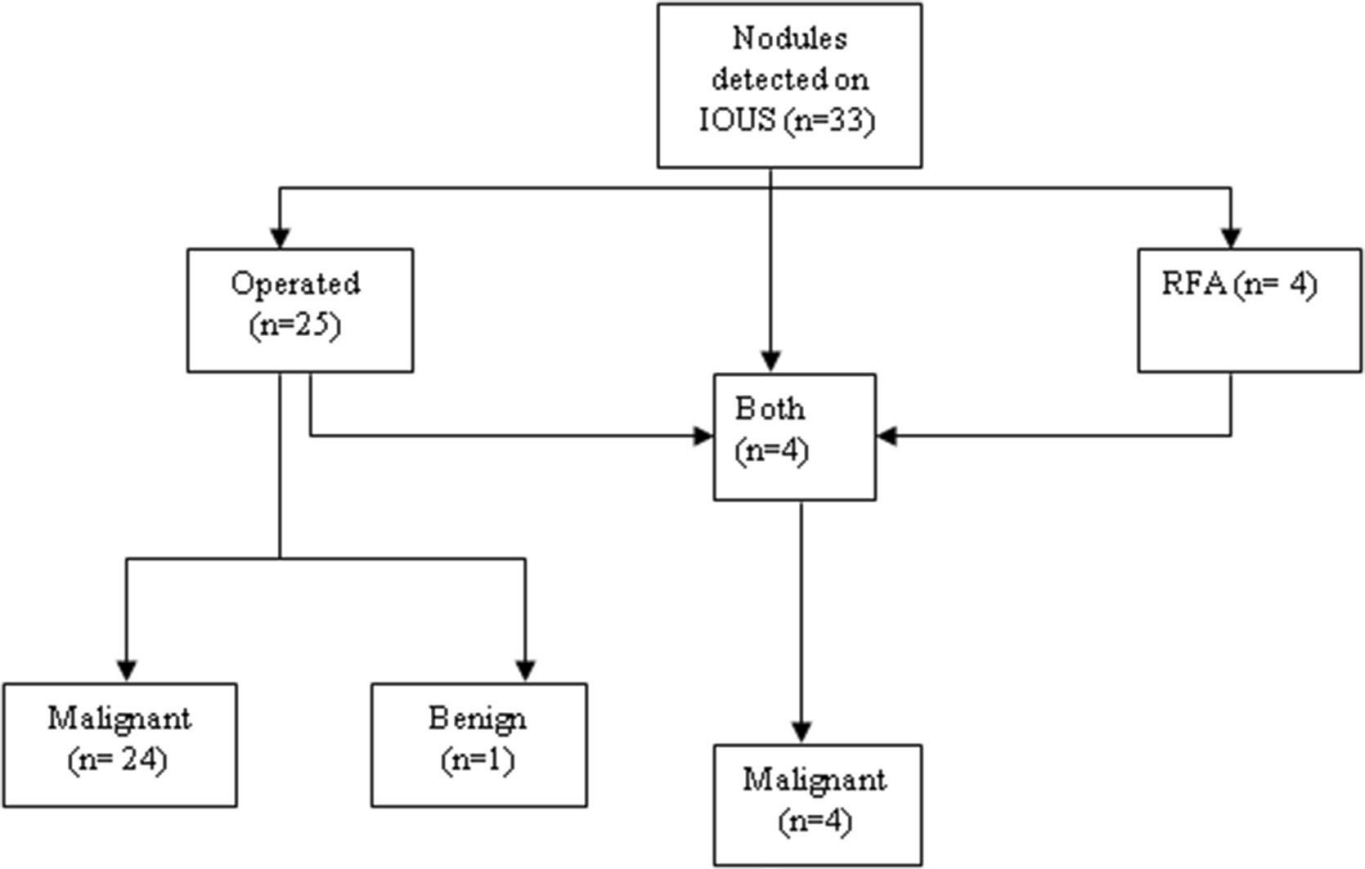
Indeterminate nodules detected on IOUS.

After liver resection, 16 (26.7%) patients developed disease recurrence. Of 60 patients with indeterminate nodules, the nodules were classified as malignant in 38 (63.3%) and benign in 16 (26.7%) on MRI. Six (10.0%) nodules underwent radiofrequency ablation and their pathological diagnosis could not be established. Indeterminate nodules were visible on IOUS in 33 (55.0%) patients and were not detected on IOUS in 27 (45.0%) patients. Of 33 patients with visible nodules on IOUS, 25 (75.8%) underwent surgical resection and four (12.1%) underwent radiofrequency ablation. The remaining four (12.1%) patients had more than one indeterminate nodule, which were both resected and ablated. Of 29 patients who underwent surgical resection, 28 (96.6%) were confirmed to be pathologically malignant. This accounts for the high positive predictive value and specificity of IOUS (Fig. 2).

**Figure 3:**
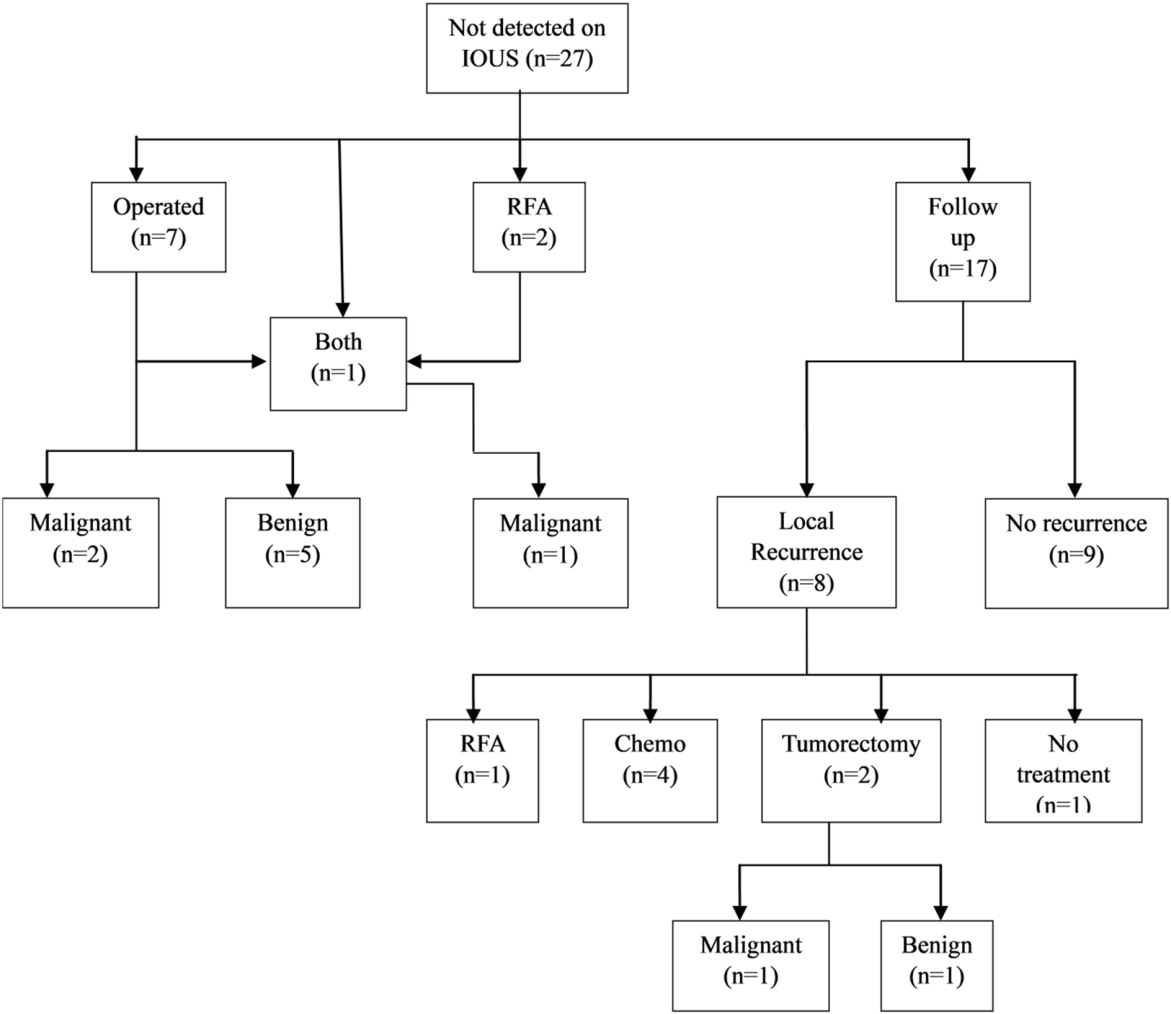
Indeterminate nodules not detected on IOUS.

Of 27 patients whose indeterminate nodules were not detected by IOUS, 17 (63.0%) were followed up while the others underwent radiofrequency ablation or unintentional surgery. Among seven patients who underwent resection, the nodules were pathologically malignant in two (28.6%) patients. Majority of the patients followed up were finally diagnosed as having benign nodules (10/17; 58.8%). Recurrence was detected by follow-up imaging in eight (47.0%) patients. two of whom underwent repeat surgery for the recurrence, and the nodule was confirmed to be pathologically benign in one patient (Fig. 3). A few patients had extrahepatic metastasis to the lung (n = 4) and lymph nodes (n = 1).

We next assessed which clinicopathological factors might predict the risk of malignant indeterminate nodules. However, only the ratio of positive lymph nodes to the total number of resected lymph nodes (pLNR) in the primary was significantly associated with the risk of malignant indeterminate nodules (*P* = 0.006; Table 2).

**Table 2:** Comparison of clinicopathological characteristics between patients with benign or malignant nodules in the follow up group.

Characteristics	Benign n = 10(%)	Malignant n = 7(%)	P value
Age > 65	5 (50)	3 (43)	0.772
> 5 nodes positive	2 (20)	3 (43)	0.309
Ratio of number of nodes positive vs number of nodes resected > 0.15	0	4 (57)	0.006
Males	7 (70)	5 (71)	0.949
Anatomical liver resection	5 (50)	2 (29)	0.377
Bilobar	4 (40)	2 (29)	0.627
Lymphatic invasion	5 (50)	5 (71)	0.377
Perineural invasion	4 (40)	4 (57)	0.486
Venous invasion	4 (40)	4 (57)	0.486
Lymphatic + Venous + Perineural invasion	1 (10)	4 (57)	0.036
Colon cancer vs rectal cancer	7 (70)	6 (86)	0.452
Synchronous lesions	5 (50)	5 (71)	0.377
CEA at diagnosis of the primary > 5	7 (70)	43 (43)	0.263
Largest size of nodule > 3 cm	3/7 (43)	2/3 (67)	0.490
No of nodules on MRI > 3	5 (50)	4 (57)	0.772
Solitary indeterminate lesions	9 (90)	6 (86)	0.787
Liver margin < 2 mm	6 (60)	5 (71)	0.627
Overall survival < 37 months	4 (40)	6 (86)	0.059

*MRI* magnetic resonance imaging, *CEA* carcinoembryonic antigen.

## Discussion

Although CT is the most common imaging modality to screen patients with CLRM, there is increasing evidence to show that MRI with hepatocyte-specific tissue contrast is better to detect small lesions characterized as indeterminate on CT with a positive predictive value of 91%^12^. A European study showed that MRI was necessary to characterize small equivocal lesions detected by CT better^14^. Besides its use for detecting CRLM, gadoxetic acid-enhanced MRI is also associated with improving the diagnostic accuracy of hepatocellular carcinoma (HCC) by detecting small HCC lesions and precursors of HCC progression^15^.

CT shows poor sensitivity for the diagnosis of lesions of < 10 mm, although its sensitivity increases with the size of the nodules^16^. In our center, gadoxetic acid-enhanced MRI is routinely performed if liver metastasis is detected by CT. Besides showing greater accuracy for the diagnosis of indeterminate nodules found on CT, MRI revealed new lesions in 138/389 patients (35.5%). The incidence of indeterminate lesions on MRI was 15.4% at our institute. Solitary indeterminate lesions were detected in 43 (71.7%) patients.

IOUS showed indeterminate nodules in 33 (55.0%) patients but no indeterminate nodules in 27 (45.0%) patients. Intriguingly, among patients in whom nodules were detected by IOUS, 96% of patients had malignant nodules, which explained the high specificity and positive predictive value of IOUS for detecting indeterminate nodules (93.75% and 96.6%, respectively). The high performance of IOUS may be due to multiple factors. First, there is no interval between IOUS and surgery, and second the operator is not blinded to the preoperative imaging and can take advantage of direct visualization of capsular lesions^17^. In a recent study in Italy, IOUS showed a higher sensitivity and specificity than hepatocyte-specific MRI for the diagnosis of new lesions and improved staging, which influenced overall and disease-free survival^18^. Although the sensitivity and specificity of IOUS were reported to be as high as 99.1% and 98.5%, respectively, in prior studies^19,20^, there are no reports of its clinical value for detecting small indeterminate lesions. Thus, we propose that IOUS should be used as an adjunct to preoperative imaging techniques to improve the staging of CRLM and thereby help select the most appropriate treatment.

Among patients whose indeterminate nodules were not detected by IOUS, 17 (63.0%) were followed up while the others underwent radiofrequency ablation or the lesions were resected unintentionally. Of seven patients who underwent resection, two (28.6%) were diagnosed with malignant nodules. Over half of the patients who were followed up had benign nodules (10/17; 58.8%). Recurrence was detected by imaging in eight (47.0%) patients. Two of these patients underwent repeat surgery for the recurrence, of which one had benign nodules.

We attempted to identify any differences in clinicopathological variables among patients with malignant nodules on follow up. However, we found no significant differences caused by the following factors: sex; age > 65 years; carcinoembryonic antigen > 5 ng/mL; more than three lesions detected by initial MRI; largest lesion of > 3 cm on MRI, lymphatic, perineural or venous invasion at primary surgery; presence of colonic or rectal metastasis; and more than five positive lymph nodes at primary surgery. The size and number of malignant nodules were not associated with malignancy in indeterminate nodules. However, the pLNR was significantly greater in patients with malignant nodules than in patients with benign nodules (*P* = 0.006). Therefore, we suggest that patients with a high pLNR after primary surgery should undergo surgical resection of indeterminate nodules, regardless of whether they are visible on IOUS or not. Recently, it was reported that the pLNR is significantly associated negatively with overall and disease-free survival^21^. The strength of pLNR is in the combination of both parameters (number of positive lymph nodes and the total number of resected lymph nodes) and was reported to be a better prognostic factor than N staging alone^22^. In a study of 295 patients in Scotland, the total number of lymph nodes retrieved and the total number of negative lymph nodes were not associated with overall survival in either colon or rectal cancers. However, in multivariable analysis, the pLNR was an independent predictor of overall survival in patients with colon cancer (hazard ratio, 11.65; 95% confidence interval, 5.00–27.15; *P* < 0.001) or rectal cancer (hazard ratio, 13.40; 95% confidence interval, 3.64–49.10; *P* < 0.001)^23^. The pLNR was reported an independent predictor for 3-year disease-free survival and overall survival in patients with CRLM who underwent curative resection and its prognostic value was superior to that of N stage and lymph node distribution^24^. Another interesting report was that the pLNR has also shown to predict patients who are at greater risk of developing metachronous CRLMs^25^.

There are some limitations to our study. First, it was a retrospective study with interobserver variation in MRI and IOUS. Second, we only included patients who underwent surgery for CRLM. Patients who have been purely followed up by the colorectal team or oncologist were not included. This may limit the number of patients with true indeterminate nodules. There may be a large number of patients with benign indeterminate lesions who are not evaluated by hepatobiliary specialists. It will be important to include such patients to increase the sample size. Third, some lesions were ablated, which precluded pathological diagnosis.

In conclusion, although hepatocyte-specific contrast agents improve the accuracy of MRI, indeterminate lesions are found in many patients. IOUS could be used as an adjunct to preoperative investigation of indeterminate lesions because of its high positive predictive value. The pLNR could be used to help select which patients can undergo conservative therapy, at least in metachronous CRLM.
